# O3-1 Key factors for sustainable implementation of community-based physical activity promotion with a focus on individuals with social disadvantages: a systematic review with narrative synthesis

**DOI:** 10.1093/eurpub/ckac094.017

**Published:** 2022-08-29

**Authors:** Natalie Helsper, Jana Semrau, Simone Kohler, Lea Dippon, Abu-Omar Karim, Alfred Rütten, Pfeifer Klaus

**Affiliations:** Department for Sport Science and Sport, Friedrich-Alexander Universität Erlangen-Nürnberg, Erlangen, Germany

**Keywords:** sustainability, physical activity promotion, community, social disadvantage

## Abstract

**Background:**

According to the German prevention law (§ 20a SGB V) of 2016, statutory health insurances are legally bound to integrate sustainable setting-based health promotion in their activities. This includes the development of sustainable concepts for community-based physical activity promotion (cbPAP) with a focus on socially disadvantaged individuals (SDI) (e.g. individuals with low income, low social status) to improve health equity. The aim of the systematic review with a narrative synthesis is to identify context-specific factors for the sustainable implementation of cbPAP and further, to inform the project KOMBINE (German acronym for ‘Community-Based PA Promotion to Implement National Recommendations’) for a scientific and practice-based implementation of the National Recommendations for Physical Activity and Physical Activity Promotion in communities.

**Methods:**

A systematic review with narrative synthesis was conducted. The search was carried out in the databases PubMed, Scopus, CINAHL, PsycINFO/SportDiscus, and WebofScience. Searches were limited to only include studies published up until 04/2020, in English and German. Altogether, qualitative, quantitative, “mixed-method” studies, reports and reviews were considered. Articles referring to factors of sustainable implementation of community-based physical activity promotion with a whole-system approach were included. Although this was not an inclusion criterion, articles addressing accessibility for SDI were specifically taken into account. The framework by Schell et al. (2013) was used for data extraction and analysis, while the four main elements from Popay et al. (2006) were applied in the narrative synthesis process.

**Results:**

29 studies (13 qualitative, 1 quantitative, 9 “mixed-method” studies, 3 reviews, 3 reports) were included in the review. 137 references to sustainability factors were identified. According to the framework by Schell et al. (2013), factors were most frequently reported in domains of organizational levels (21), partnerships (18) and financial stability (16). Additional factors in the domains of accessibilities for SDI (11), infrastructure (7), participation (9), and cultural context (10) were identified by the authors. The relationship between the factors and the context of the programs are considered as well.

**Conclusions:**

The identified factors for sustainability should be systematically addressed at an early stage in the conception of cbPAP measures for SDI to ensure sustainability after implementation.

